# Opinion: Treat

**DOI:** 10.1590/S1677-5538.IBJU.2016.02.03

**Published:** 2016

**Authors:** Eduardo Mazzuchi

**Affiliations:** 1 Divisão de Urologia do Hospital das Clínicas da Universidade de São Paulo Faculdade de Medicina de São Paulo, Brasil

**Keywords:** Urinary Calculi, Kidney Calculi, Hydronephrosis

The prevalence of urinary stones is increasing and reached 8.8% in the United States in 2010 (1). We do not have statistics on this issue in developing countries but the impression is that this is a worldwide tendency thanks to an increase in obesity and to our modern lifestyle. The proportion of asymptomatic renal stones is also increasing probably due to the more frequent use of image methods. According to the literature 20 to 44% of renal stones are located in the lower pole and to treat them or not and how to do that has been motive of debate (2, 3). The importance of this issue is capital once almost every day we are asked to see a completely asymptomatic and surprised patient with an ultrasound done for any other reason showing a small solitary caliceal lower pole stone.

Many articles on this issue are retrospective with a low number of patients and short follow-up (2). Due to the scarcity of large populational studies the European Association of Urology issued a grade C recommendation that asymptomatic renal stones can be followed up yearly for the first two or three years while intervention should be considered after this period and the American Urological Association has not yet released a statement on this issue (2, 4).

Contemporary articles in the literature report 11 to 20% spontaneous elimination of caliceal lower pole stones (with or without pain) (2, 4, 5). Progression (i.e. growing, infection or pain) occur in 33 to 46% of patients and the urge for surgery in 3 to 19% of patients and seem to be higher among bigger stones (2, 4–6). In a two-year follow-up 90% remained asymptomatic according to Inci at al (5). According to a recent article published in The Journal of Urology by Dropkin et al., in a follow-up period of 3.5 years performed with ultrasound every six months lower pole caliceal stones caused symptoms in 24% of patients compared to 40% of asymptomatic non lower pole stones. Spontaneous passage occurred in 3% against 14.5% of non-lower pole stones and surgery for removal was necessary in 18.6% in the first group and 20.3% in the second. Additionally 2% of the stones caused silent hydronephrosis leading to surgical treatment. According to these data, the need for surgery is small and less than 50% of patients showed progression of their disease during the follow-up period. Based on this and in other studies active surveillance may be a safe option for non-obstructing asymptomatic lower pole caliceal stones.

However some problems may arise when active surveillance for a potentially non-lethal disease is to be implemented in a developing country. We all know that in many regions the access to a more specialized medicine is limited, especially in rural areas due to difficult transportation or absence of proper medical services. Performing an appropriate follow-up requires at least an ultrasound device, personnel with expertise in the field and some basic laboratory analysis which we all know are not easily available worldwide. As stone disease is not considered a priority in our and many other medical systems it is difficult to believe that long-term follow-up is possible in every parts of many countries. As mentioned before in 2% of the cases silent hydronephrosis developed and surgical treatment was required. One can imagine that without proper follow-up some kidneys can be lost if this complication occur. Additionally the majority of articles state that progression is linked to the size of the stone and we do not know exactly if stones smaller than 5 mm have better outcomes than those with 5-10 mm. The spontaneous passage rate of stones ≤ 5mm is higher than 5 to 10mm stones and some authors believe that stones bigger than 5 mm should always be treated (7). Another point is that the great majority of the published articles do not approach some special groups of patients like solitaire kidney patients, immunocompromised patients, professional categories like air pilots, people travelling frequently to remote areas or women intending to get pregnant soon among others.

We all know that the majority of patients seek doctor's recommendation in a shared decision model. This way following patients regularly with small asymptomatic lower pole stones is a good alternative if three basics conditions are fulfilled: 1. your patient is motivated to do it; 2. you have technical condition to do it properly which means to perform at least an ultrasound and clinical evaluation every six months or once a year; 3. your patient is not included in one of the groups mentioned above or have any other particular medical condition. Otherwise removing the stone may be the best alternative!
